# Synthesis of Cyanate Esters Based on Mono-*O*-Methylated Bisphenols with Sulfur-Containing Bridges

**DOI:** 10.3390/molecules24010177

**Published:** 2019-01-04

**Authors:** Andrey Galukhin, Roman Nosov

**Affiliations:** Kazan Federal University, 18 Kremlevskaya Street, 420008 Kazan, Russia; romanosow@mail.ru

**Keywords:** cyanate esters, aryl cyanates, cyanate resins, curing, bisphenols, Mitsunobu reaction, thermal analysis

## Abstract

We described a synthetic approach to bisphenol-based monocyanate esters based on mono-*O*-methylation of parental bisphenols followed by cyanation of the residual phenolic hydroxyl. Structures of the synthesized compounds were determined by the application of IR, NMR ^1^H and ^13^C spectroscopies, EI and MALDI mass spectrometry, and purity of the final product was controlled by HPLC. We showed that stability of the cyanate esters depends on the nature of the bridging group. Temperature range of thermally initiated cyclotrimerization of synthesized monocyanate ester, as well as reaction enthalpy, was determined by differential scanning calorimetry (DSC).

## 1. Introduction

**Figure 1:**
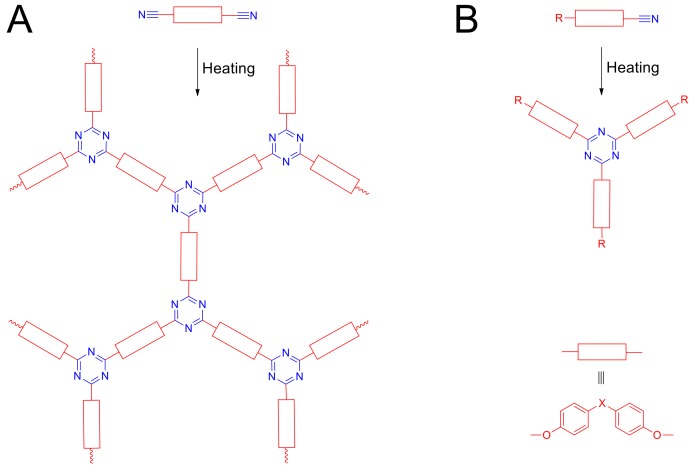
Schemes of polycyclotrimerization of dicyanate esters (**A**) and cyclotrimerization of monocyanate esters (**B**).

The outstanding properties of cyanate resins (Figure 1), such as mechanical properties, thermal properties (high glass transition temperature, high char yield), electrical properties (low dielectric loses, low dissipation factors), high hydrolytic stability and low water absorption, make them irreplaceable for electronic, aerospace and military industry [1]. The molding process of cyanate resins requires a choice of appropriate temperature program, thus the kinetics of the network-forming reaction is demanded [2,3,4,5]. The heating of cyanate esters containing two or more cyanate groups leads to polycyclotrimerization reaction resulting in formation of cross-linked polymer network (Figure 1A). In general, the kinetics of cross-linking is often complicated by diffusion due to the raise of viscosity (several orders of magnitude) of the cured system even at low conversions [6], thus to study the kinetics of chemical reaction in detail it is necessary to exclude significant raise of viscosity of the cured reaction mixture.

To resolve this task we decided to use model monocyanate esters, which is known to undergo simple cyclotrimerization (Figure 1B) without formation of cross-linked polymeric network [7]. Since the commercially available cyanate esters mostly presented by derivatives of bisphenols of all kind, to obtain target compounds, we decided to protect one phenolic hydroxyl group of the parental bisphenol by non-reacting group, followed by further transformation of residual phenolic hydroxyl to monocyanate ester. Heating of compound of such structure leads to simple cyclotrimerization product, so chemical kinetics of the reaction might be studied in detail.

Thus, the main objective of this study is to develop the approach to synthesis of monocyanate esters based on consecutive mono-*O*-methylation of bisphenols by means of Mitsunobu synthesis followed by cyanation reaction.

## 2. Results and Discussion

### 2.1. Synthesis and Characterization of Target Compounds

**Figure 2:**
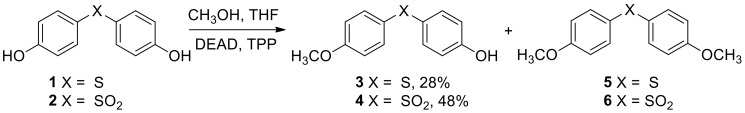
Synthetic route to mono-*O*-methylated bisphenols **3** and **4**.

We decided to use well known Mitsunobu synthesis [8] to obtain mono-*O*-methylated derivatives of bisphenols **1** and **2** (Figure 2). This synthetic approach allows esterification to proceed fast and in very mild conditions. 

It should be noted, that synthesis of target compounds **3** and **4** was accompanied by formation of substantial amount of dimethylated by-products **5** and **6,** even at equimolar ratio of bisphenol to methanol. We assume that reactivity of monomethylated products **3** and **4** in Mitsunobu reaction is higher than that of parental bisphenols **1** and **2**, which results in quite low yields of target compounds. The structures of the products **3** and **4** were confirmed by a combination of IR, ^1^H, ^13^C NMR spectroscopies and electron ionization mass spectrometry (Figures S1–S8 in Supplementary Information).

**Figure 3:**
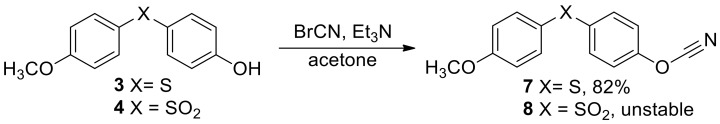
Synthetic route to monocyanate esters **7** and **8**.

The next step was conversion of the obtained mono-*O*-methylated compounds **3** and **4** into subsequent cyanate esters **7** and **8**. The most common and convenient way for such synthesis is nucleophilic substitution by BrCN in presence of organic bases [9] (Figure 3). An alternative approaches to synthesis of cyanate esters using thermolysis of thiathriazoles [10] and decomposition of thiocarbamates on heavy metal oxides [11] give much lower yields of target products and therefore were not applied.

The formation of target compounds **7** was confirmed by a combination of spectroscopic methods and mass spectrometry (Figures S9–S12 in Supplementary Information). The most demonstrative confirmation of formation of target compound **7**, besides mass spectrometry, was IR spectroscopy: the disappearance of absorption band of 3400 cm^−1^ related to OH phenolic group and appearance of absorption bands in a region of 2200–2400 cm^−1^ evidence about the presence of OCN group into molecule.

It should be noted compound **8** was hydrolytically unstable and cannot be obtained with desirable purity: contact with ice-cold water or even water vapors results in its decomposition to parental phenol **4**. Silanol groups of silica gel also catalyze that process, thus usual chromatographic purification procedure cannot be applied to that particular compound. We assume the most probable reason for such instability of compound **8** is influence of electron-withdrawing SO_2_ bridge group, which increase stability of corresponding phenolate anion and thus promotes its formation through hydrolysis of compound **8**.

**Figure 4:**
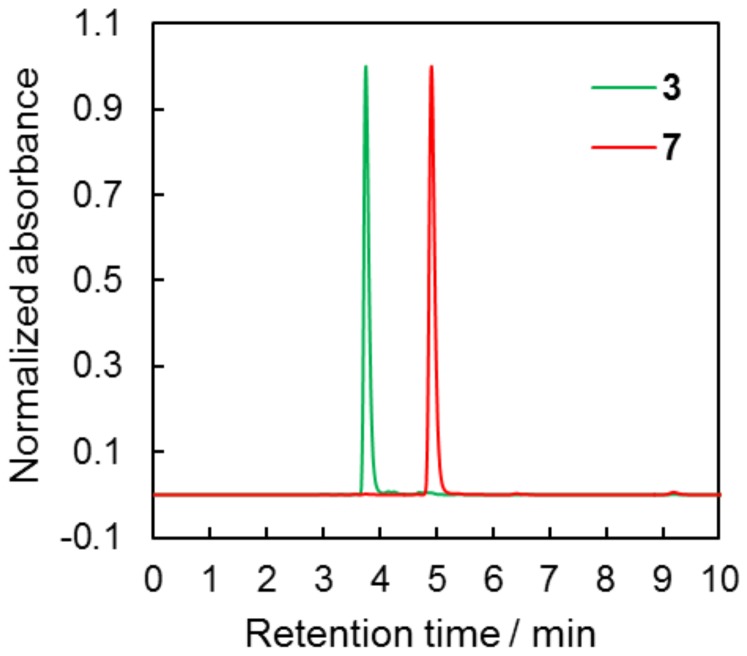
Chromatograms of compounds **3** and **7**.

Since the reaction of aryl cyanates polymerization is sensitive to nucleophilic compounds [11,12,13,14], as with residual phenols, the purity of the obtained aryl cyanate **7** was a concern. We evaluated purity of that compound by HPLC. Figure 4 shows chromatograms for compound **7** monomer and parental phenol **3**. The retention times for compounds **3** and **7** were 3.75 and 4.91 min subsequently.

It should be stressed obtained after purification monomer did not contain parental phenol in quantities detectable by HPLC and its purity was not less than 99.5%.

### 2.2. Differential Scanning Calorimetry Study

**Figure 5:**
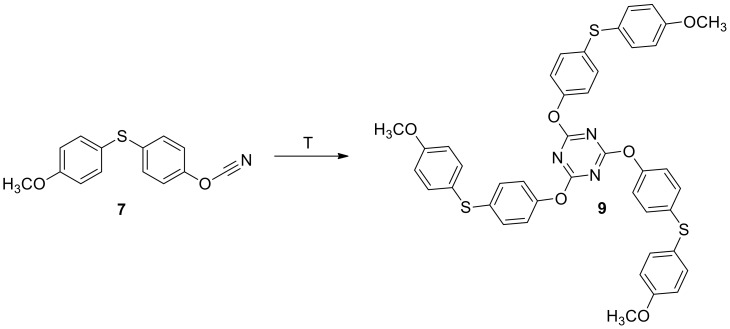
Scheme of cyclotrimerization of compound **7**.

As it was mentioned before cyanate esters containing single cyanate group undergo thermo-initiated exothermic cyclotrimerization upon heating (Figure 5). We used differential scanning calorimetry (DSC) to determine enthalpy of cyclotrimerization and study temperature intervals where curing occurred. 

**Figure 6:**
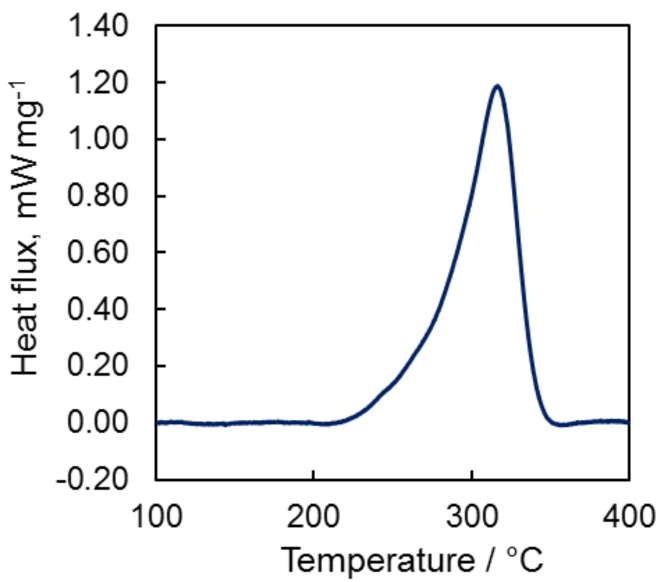
DSC curve for cyclotrotrimerization of compound **7** (heating rate of 10 °C min^−1^, argon atmosphere).

Figure 6 shows DSC curve for compound and it might be seen that cyclotrimerization process proceeds in temperature range of 200–350 °C (for heating rate of 10 °C min^−1^). The enthalpy of cyclotrimerization for compound **7** was determined as 350 ± 20 J g^−1^ or 90 ± 5 kJ per 1 mole of cyanate groups; obtained value is in a good agreement with the literature ones [1,3,13,14] reported for compounds of similar structure.

**Figure 7:**
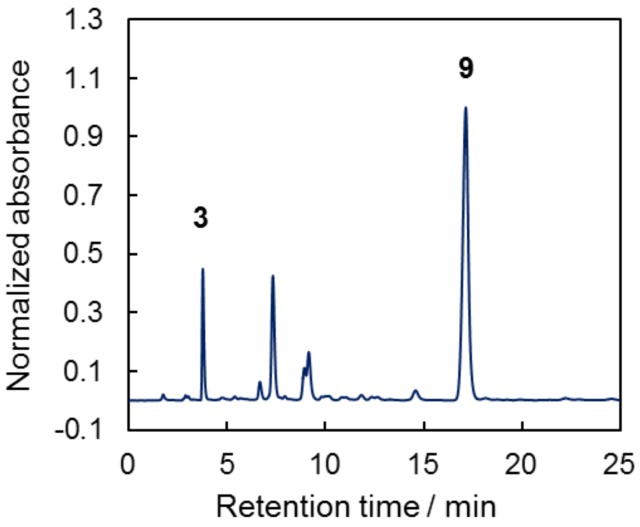
Chromatogram of curing product of **7**.

We also studied the composition of the product formed during curing of cyanate ester 7. For that purpose, the DSC pan containing 8 mg of the compound **7** was heated to 340 °C at a heating rate of 10 °C min^−1^, then cooled and composition of the obtained product was analyzed by HPLC (Figure 7). The first peak on chromatogram correspond to initial compound **3** (retention time is 3.76 min which matches with 3.75 min obtained for pure compound), and the most intense peak presumably correspond to final product of cyclotrimerization **9**, containing 1,3,5-triazine fragment, that gives most intense peak in MALDI mass spectrum with *m*/*z* = 772.8, which correspond to [M + H]^+^ ion of the curing product **9**. 

**Figure 8:**
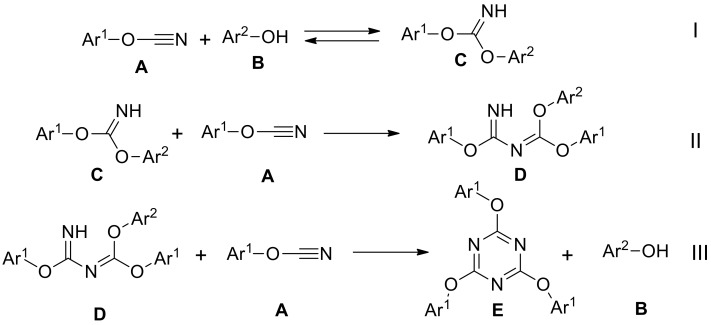
Mechanism of aryl cyanates cyclotrimerization.

The presence of other signal on chromatogram can be explained by complex mechanism of cyanate esters cyclotrimerization, which includes three main steps I–III [15] (Figure 8). The first step is commonly described by reaction of aryl cyanate **A** with nucleophiles, such as phenol **B**, resulting in formation of imidocarbonate **C**. The reaction I can be considered as a reversible based on existing literature data [9]. Second (II) and third (III) steps involve consecutive addition of two cyanates with formation of cyclic product **E** containing 1,3,5-triazine ring.

Thus, other signals on chromatogram might be attributed to formation of intermediates, structure of which might be determined after their separation in pure form and analyzing their spectroscopic parameters, which is a theme of our future study.

## 3. Materials and Methods 

### 3.1. Materials

4,4′-Thiodiphenol (>98%, TCI), 4,4′-sulfonyldiphenol (99.7%, Acros Organics), diethyl azodicarboxylate (DEAD, 40% solution in toluene, Aldrich Chemistry, Saint Louis, MI, USA), triphenylphosphine (TPP, 99%, Sigma-Aldrich, Saint Louis, MI, USA), triethylamine (>99%, Sigma-Aldrich, Saint Louis, MI, USA), cyanogen bromide (97%, Acros Organics, New Jersy, NJ, USA), acetone (extra pure, Acros Organics, New Jersy, NJ, USA), trichlormethane (>99.5%), dichlormethane (>99.5%), tetrahydrofuran (THF, >99.5%), ZnCl_2_ (>99.5%), Na_2_SO_4_ (anhydrous, >99.5%), SiO_2_ (60 Å, 0.04–0.063 mm, Machery-Nagel, Bethlehem, PA, USA). All reagents were purchased and used without additional purification. Deionized water (18.2 MΩ) was obtained by Arium mini instrument (Sartorius, Gottingen, Germany).

### 3.2. Methods

Column chromatography was performed with slurry packed silica gel.

The ^1^H, ^13^C NMR spectra were recorded on Bruker Avance-400 spectrometer. Chemical shifts were determined relatively to the signals of residual protons of the deuterated solvent (CDCl_3_). Chemical shifts are reported in delta (δ) units in parts per million (ppm) and splitting patterns are designated as s, singlet; d, doublet; t, triplet; q, quartet; m, multiplet and br, broad. Coupling constants are recorded in Hertz (Hz).

IR spectra were recorded with Bruker Vertex 70 FTIR spectrometer with a single reflection, germanium crystal attenuated total reflectance accessory (MIRacle, PIKE Technologies, Fitchburg, WI, USA). The interferograms were recorded with a resolution of 2 cm^−1^, 128 scans, and Fourier transformed using a Blackman-Harris apodization function.

EI spectra were recorded by 5977b MSD instrument (Agilent Technologies, Santa Clara, CA, USA). MALDI spectra were recorded by Bruker autoflex speed mass-spectrometer using dithranol or 2,5-dihydroxybenzoic acid as the matrix.

The HPLC analyzes were carried out with a Dionex Ultimate 3000 chromatograph equipped with a UV detector (254 nm) and Dionex Acclaim 120 chromatographic column (C18-bonded silica, 5 µm, 120 Å, 4.6 × 250 mm). Mixture of 86% of acetonitrile and 14% of deionized water (by volume) was used as an eluent at a flow rate of 1 mL min^−1^.

All calorimetric measurements were carried out using thermoanalyzer STA 449 F1 Jupiter (Netzsch) in temperature range of 100–400 °С. The experiments were conducted at linear heating rate of 10 °С min^-1^ under argon flow (75 mL min^−1^) in 40 µL aluminum sealed pans. Pans were sealed in argon flushed glovebox equipped by beaker with P_2_O_5_ to prevent water adsorption by cyanate esters. The mass of the sample for each run was 4 mg.

### 3.3. Synthesis Details and Characterization

#### 3.3.1. Synthesis and Characterization of 4-((4′-methoxyphenyl)thio)phenol **3**

In a 100 mL round bottom flask equipped by magnetic stirrer 3 g (13.7 mmol) of 4,4’-thiodiphenol, 0.56 mL (13.7 mmol) of methanol, 3.61 g (13.7 mmol) of TPP and 20 mL THF were added. Obtained reaction mixture was cooled in ice-bath and 5.9 g of DEAD (40% solution in toluene) was added dropwise. Then reaction mixture was stirred for 12 h at room temperature. Then excess of ZnCl_2_ solution in ethyl acetate was added to reaction mixture to bind triphenylphosphine oxide (TPPO) and white crystalline precipitate of ZnCl_2_-TPPO was removed by filtration. Then solvent was removed from filtrate under reduced pressure and obtained brown oil was chromatographed in trichloromethane. Obtained yellow oil was dissolved in 50 mL of benzene, subsequently washed by 20 mL 4 M NaOH and distilled water (till neutral pH) and dried over anhydrous Na_2_SO_4_. Obtained solution contained mixture of mono- and dimethylated derivatives of 4,4’-thiodiphenol, which were separated by column chromatography (dichloromethane as an eluent). 

Synthesis of 4-((4′-methoxyphenyl)thio)phenol was obtained as a white solid at a yield of 28% (0.91 g). IR (KBr/cm^−1^): 3461, 3389 (-OH). ^1^H-NMR, CDCl_3_-d1, δ (ppm): 3.79 (s, 3H, CH_3_-O), 4.97 (br.s, 1H, -OH), 6.75–6.85 (m, 4H, Ph-H), 7.21–7.29 (m, 4H, Ph-H). ^13^C-NMR, δ (ppm): 158.99, 154.92, 132.86, 127.71, 127.29, 116.23, 114.80, 55.39. EI MS analysis shows a signal at m/z = 232,1 corresponding to [M]^+^ (calc. mass for M (C_13_H_12_O_2_S): 232.05). Elemental analysis for (C_13_H_12_O_2_S). Calculated (%): C, 67.22%, H, 5.21%, S, 13.80%. Found (%): C, 67.43%, H, 5.90%, S, 13.97%.

#### 3.3.2. Synthesis and Characterization of 4-((4′-methoxyphenyl)sulfonyl)phenol **4**

In a 100 mL round bottom flask equipped by magnetic stirrer 5 g (20.0 mmol) of 4,4’-sulfonyldiphenol, 0.81 mL (20.0 mmol) of methanol, 5.24 g (20.0 mmol) of TPP and 30 mL THF were added. Obtained reaction mixture was cooled in ice-bath and 8.7 g of DEAD (40% solution in toluene) was added there dropwise. Then reaction mixture was stirred for 12 h at room temperature. Then excess of ZnCl_2_ solution in ethyl acetate was added to reaction mixture to bind triphenylphosphine oxide (TPPO) and white crystalline precipitate of ZnCl_2_-TPPO was removed by filtration. Then solvent was removed from filtrate under reduced pressure and obtained oily residue was dissolved in benzene and washed by 3 × 20 mL 4 M NaOH. Water layers were collected and combined, and then 1 M hydrochloric acid solution was added till Ph ≈ 1. Obtained acidic solution was extracted by benzene 3 × 20 mL, organic fractions were collected and combined. Then solvent was removed under reduced pressure and target compound was separated by column chromatography (dichloromethane:methanol 100:1 as an eluent).

Synthesis of 4-((4-methoxyphenyl)sulfonyl)phenol was obtained as a white solid at a yield of 48% (2.55 g). IR (KBr/cm^−1^): 3431 (-OH). ^1^H-NMR, CDCl_3_-d1, δ (ppm): 3.83 (s, 3H, CH_3_-O), 6.91–6.95 (m, 4H, Ph-H), 7.42 (br.s, 1H, OH), 7.73–7.83 (m, 4H, Ph-H). ^13^C-NMR, δ (ppm): 163.29, 160.66, 133.28, 132.68, 129.60, 129.39, 116.23, 114.58, 55.69. EI MS analysis shows a signal at *m*/*z* = 264,1 corresponding to [M]^+^ (calc. mass for M (C_13_H_12_O_4_S): 264.05). Elemental analysis for (C_13_H_12_O_4_S). Calculated (%): C, 59.08%, H, 4.58%, S, 12.13%. Found (%): C, 59.24%, H, 4.71%, S, 12.18%.

#### 3.3.3. Synthesis and Characterization of (4-cyanatophenyl)(4′-methoxyphenyl)sulfane **7**

In a 100 mL round bottom flask equipped by magnetic stirrer 1.0 g (4.3 mmol) of 4-((4-methoxyphenyl)thio)phenol, 0.9 g (8.6 mmol) of cyanogen bromide and 30 mL of acetone were added. Obtained reaction mixture was cooled to −30 °C and 1.2 mL (8.6 mmol) of triethylamine was added dropwise and reaction mixture was stirred for 1 h. Then solvent was removed from reaction mixture under reduced pressure and obtained residue was dissolved in trichloromethane and washed several times with deionized water. Obtained organic solution was dried over anhydrous Na_2_SO_4_ and target compound **7** was separated by column chromatography (dichloromethane as an eluent).

Synthesis of (4-cyanatophenyl)(4-methoxyphenyl)sulfane was obtained as a white solid at a yield of 82% (0.90 g). IR (KBr/cm^−1^): 2360, 2236 (-CN). ^1^H-NMR, CDCl_3_-d1, δ (ppm): 3.92 (s, 3H, CH_3_-O), 7.00–7.52 (m, 8H, Ar-H). ^13^C-NMR, δ (ppm): 160.36, 150.84, 138.29, 135.94, 129.39, 122.94, 115.87, 115.32, 108.67, 55.44. EI MS analysis shows a signal at *m*/*z* = 257.1 corresponding to [M]^+^ (calc. mass for M (C_14_H_11_NO_2_S): 257.05). Elemental analysis for (C_14_H_11_NO_2_S). Calculated (%): C, 65.35%, H, 4.31%, S, 12.46 %. Found (%): C, 65.53%, H, 4.39%, S, 12.37%.

#### 3.3.4. Synthesis of 1-cyanato-4-((4-methoxyphenyl)sulfonyl)benzene **8**

In a 100 mL round bottom flask equipped by magnetic stirrer 1.0 g (3.8 mmol) of 4-((4-methoxyphenyl)sulfonyl)phenol, 0.8 g (7.6 mmol) of cyanogen bromide and 30 mL of acetone were added. Obtained reaction mixture was cooled to −30 °C and 1.1 mL (7.6 mmol) of triethylamine was added dropwise and reaction mixture was stirred for 1 h. Then solvent was removed from reaction mixture under reduced pressure and obtained residue was dissolved in trichloromethane and washed several times with ice-cold deionized water. Obtained organic solution was dried over anhydrous Na_2_SO_4_, filtered and then organic solvent was removed under reduced pressure.

## 4. Conclusions

In the current study we synthesized mono-*O*-methylated bisphenols and used them as precursors for synthesis of subsequent cyanate esters. It turned out S-bridged cyanate ester is readily available by proposed synthetic approach and can be obtained with a high purity, which is required for our further kinetic studies, whereas SO_2_-bridged cyanate ester is hydrolytically unstable and is readily hydrolyzed in presence of water vapors. Curing process of the obtained S-bridged cyanate ester was studied by DSC, temperature intervals of the reaction as well as reaction enthalpy were established. The composition of the curing product was partially determined and formation of cyclotrimerization product were established.

## Supplementary Materials

The following are available online, Figure S1: NMR 1H spectrum of 4-((4-methoxyphenyl)thio)phenol, Figure S2: NMR 13С spectrum of 4-((4-methoxyphenyl)thio)phenol, Figure S3: IR spectrum of 4-((4-methoxyphenyl)thio)phenol, Figure S4: EI mass spectrum of 4-((4-methoxyphenyl)thio)phenol, Figure S5: NMR 1H spectrum of 4-((4-methoxyphenyl)sulfonyl)phenol, Figure S6: NMR 13C spectrum of 4-((4-methoxyphenyl)sulfonyl)phenol, Figure S7: IR spectrum of 4-((4-methoxyphenyl)sulfonyl)phenol, Figure S8: EI mass spectrum of 4-((4-methoxyphenyl)sulfonyl)phenol, Figure S9: NMR 1H spectrum of (4-cyanatophenyl)(4-methoxyphenyl)sulfane, Figure S10: NMR 13C spectrum of (4-cyanatophenyl)(4-methoxyphenyl)sulfane, Figure S11: IR spectrum of (4-cyanatophenyl)(4-methoxyphenyl)sulfane, Figure S12: EI mass spectrum of (4-cyanatophenyl)(4-methoxyphenyl)sulfane, Figure S13: MALDI mass spectrum of (4-cyanatophenyl)(4-methoxyphenyl)sulfane cyclotrimerization product.
