# Supplementary material for: Synthesis of Cyanate Esters Based on Mono-O-Methylated Bisphenols with Sulfur-Containing Bridges
Source: Molecules. 2019 Jan 4;24(1):177. doi: 10.3390/molecules24010177 (PMC6337567; doi:10.3390/molecules24010177)

Synthesis of cyanate esters based on mono-*O*-methylated bisphenols with sulfur-containing bridges

Andrey Galukhin*, Roman Nosov

[1] Kazan Federal University, 29/1 Lobachevskogo Street, 420008, Kazan, Russia
* Correspondence: and_galuhin@mail.ru; Tel.: +7917-275-8404

Table of Contents:

Figure S1. NMR ^{1}H spectrum of 4-((4-methoxyphenyl)thio)phenol

Figure S2. NMR ^{13}C spectrum of 4-((4-methoxyphenyl)thio)phenol

Figure S3. IR spectrum of 4-((4-methoxyphenyl)thio)phenol

Figure S4. EI mass spectrum of 4-((4-methoxyphenyl)thio)phenol

Figure S5. NMR ^{1}H spectrum of 4-((4-methoxyphenyl)sulfonyl)phenol

Figure S6. NMR ^{13}C spectrum of 4-((4-methoxyphenyl)sulfonyl)phenol

Figure S7. IR spectrum of 4-((4-methoxyphenyl)sulfonyl)phenol

Figure S8. EI mass spectrum of 4-((4-methoxyphenyl)sulfonyl)phenol

Figure S9. NMR ^{1}H spectrum of (4-cyanatophenyl)(4-methoxyphenyl)sulfane

Figure S10. NMR ^{13}C spectrum of (4-cyanatophenyl)(4-methoxyphenyl)sulfane

Figure S11. IR spectrum of (4-cyanatophenyl)(4-methoxyphenyl)sulfane

Figure S12. EI mass spectrum of (4-cyanatophenyl)(4-methoxyphenyl)sulfane

Figure S13. MALDI mass spectrum of (4-cyanatophenyl)(4-methoxyphenyl)sulfane cyclotrimerization product

Figure S1. NMR ^{1}H spectrum of 4-((4-methoxyphenyl)thio)phenol

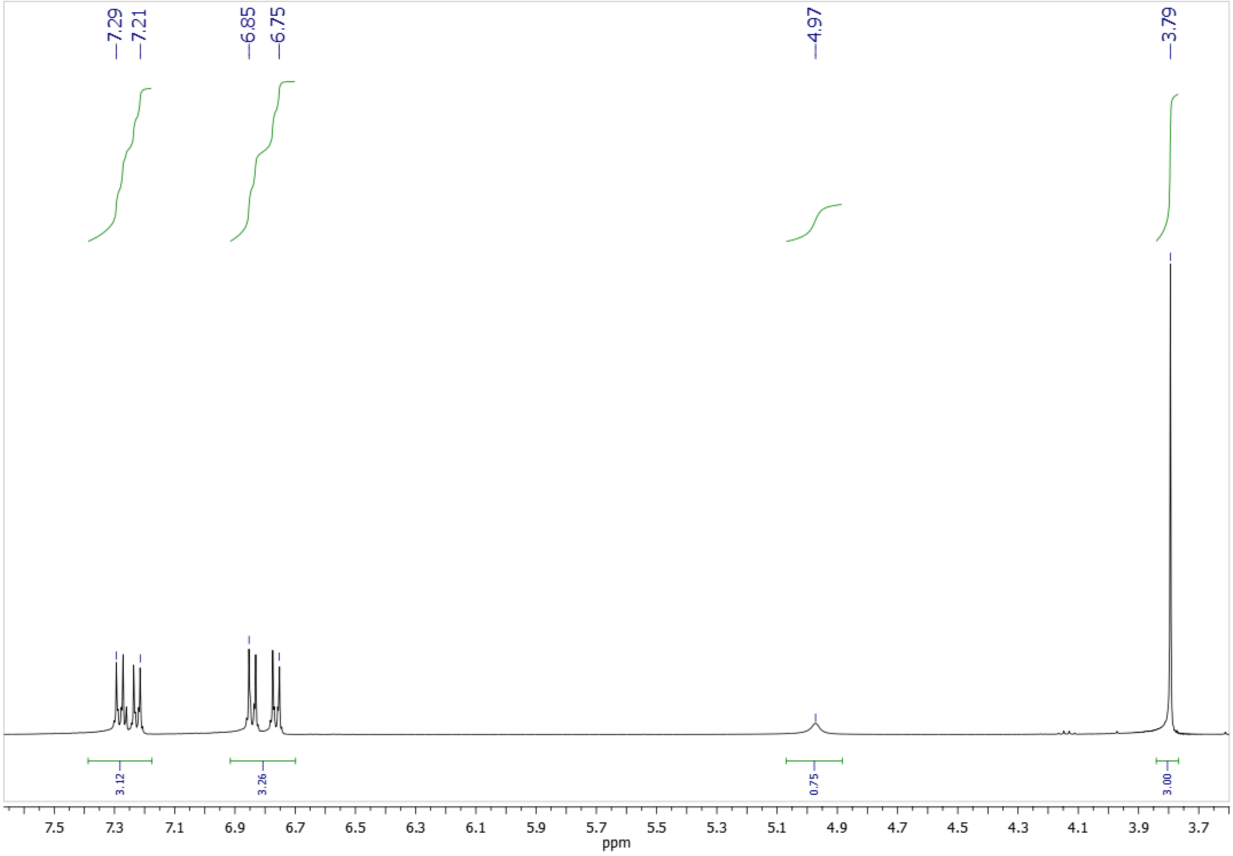

Figure S2. NMR ^{13}C spectrum of 4-((4-methoxyphenyl)thio)phenol

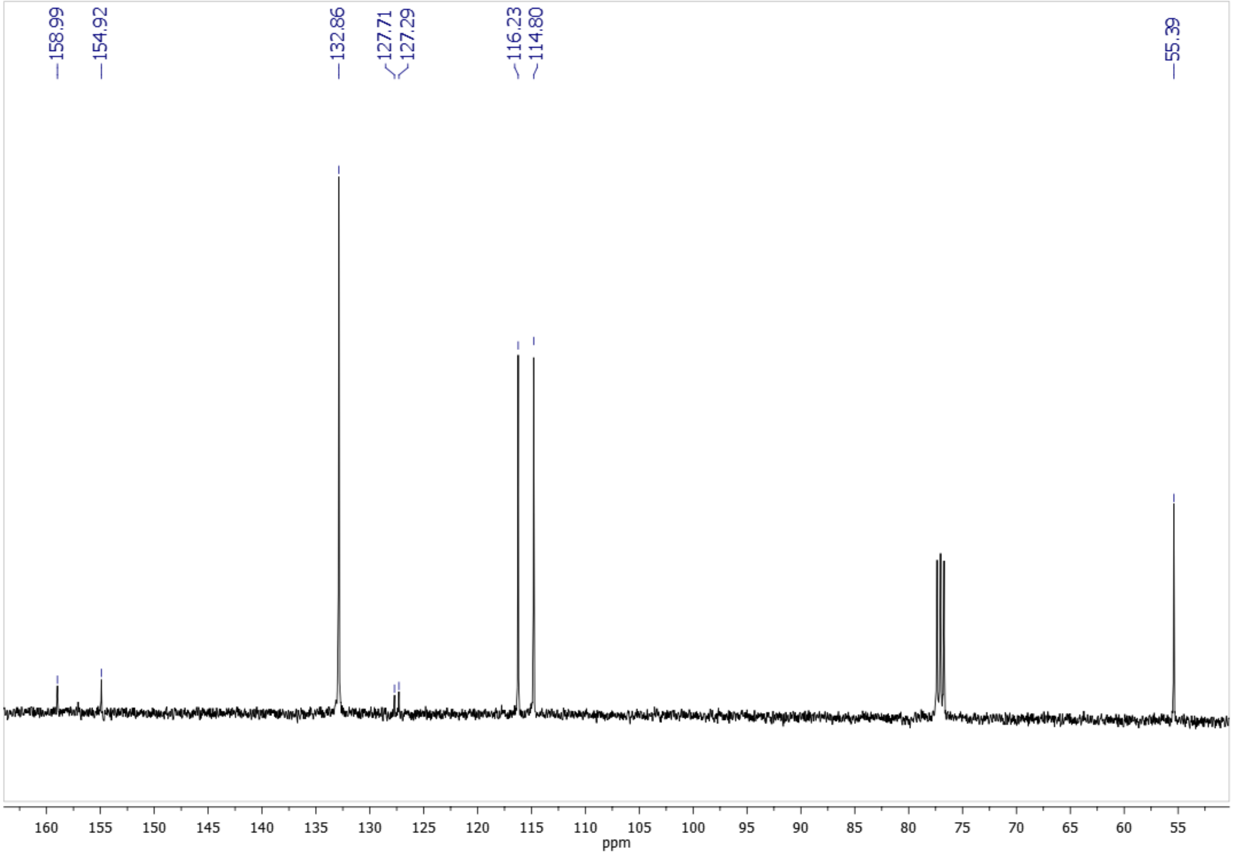

Figure S3. IR spectrum of 4-((4-methoxyphenyl)thio)phenol

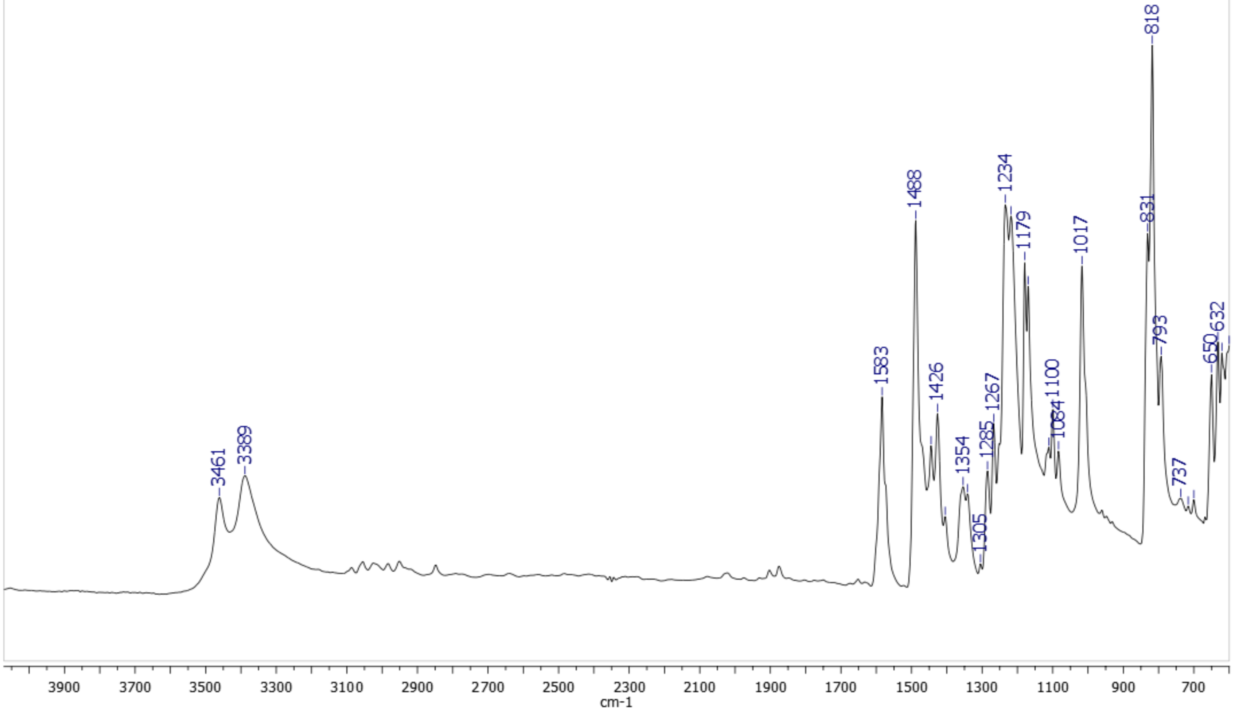

Figure S4. EI mass spectrum of 4-((4-methoxyphenyl)thio)phenol

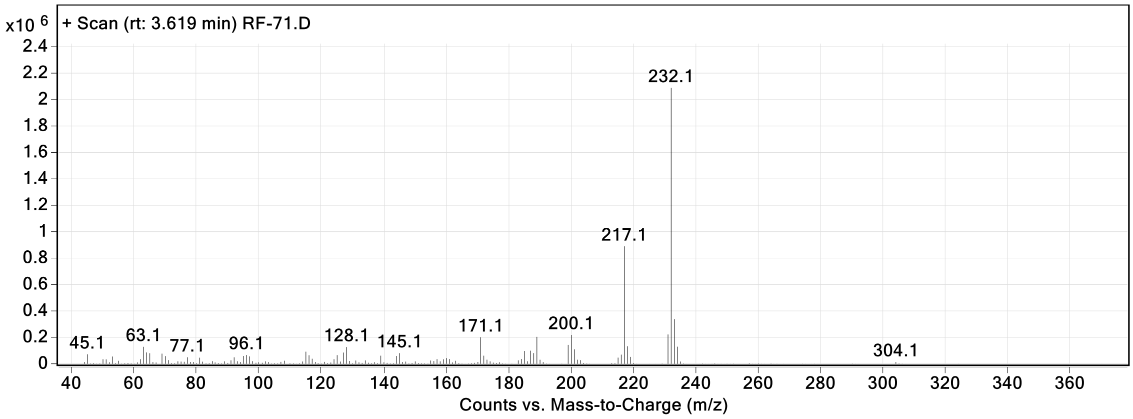

Figure S5. NMR ^{1}H spectrum of 4-((4-methoxyphenyl)sulfonyl)phenol

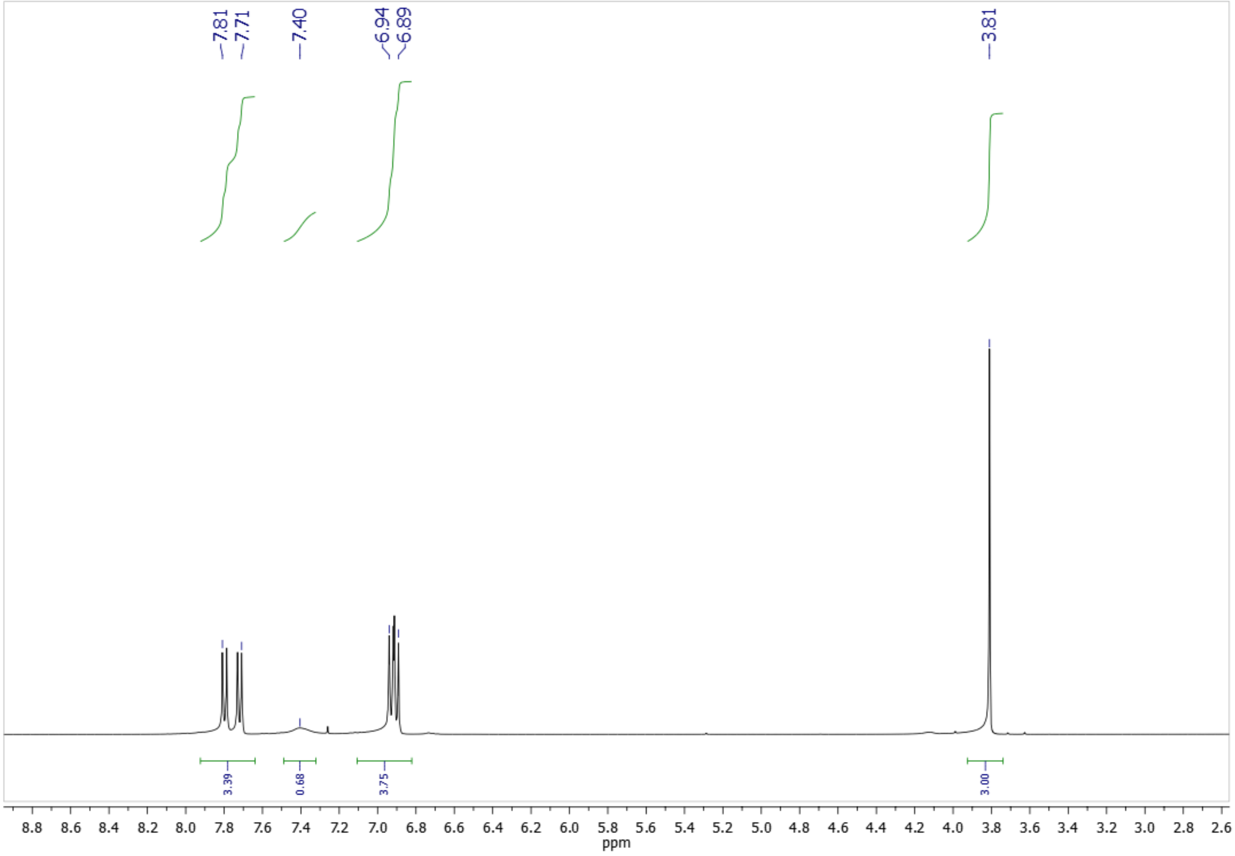

Figure S6. NMR ^{13}C spectrum of 4-((4-methoxyphenyl)sulfonyl)phenol

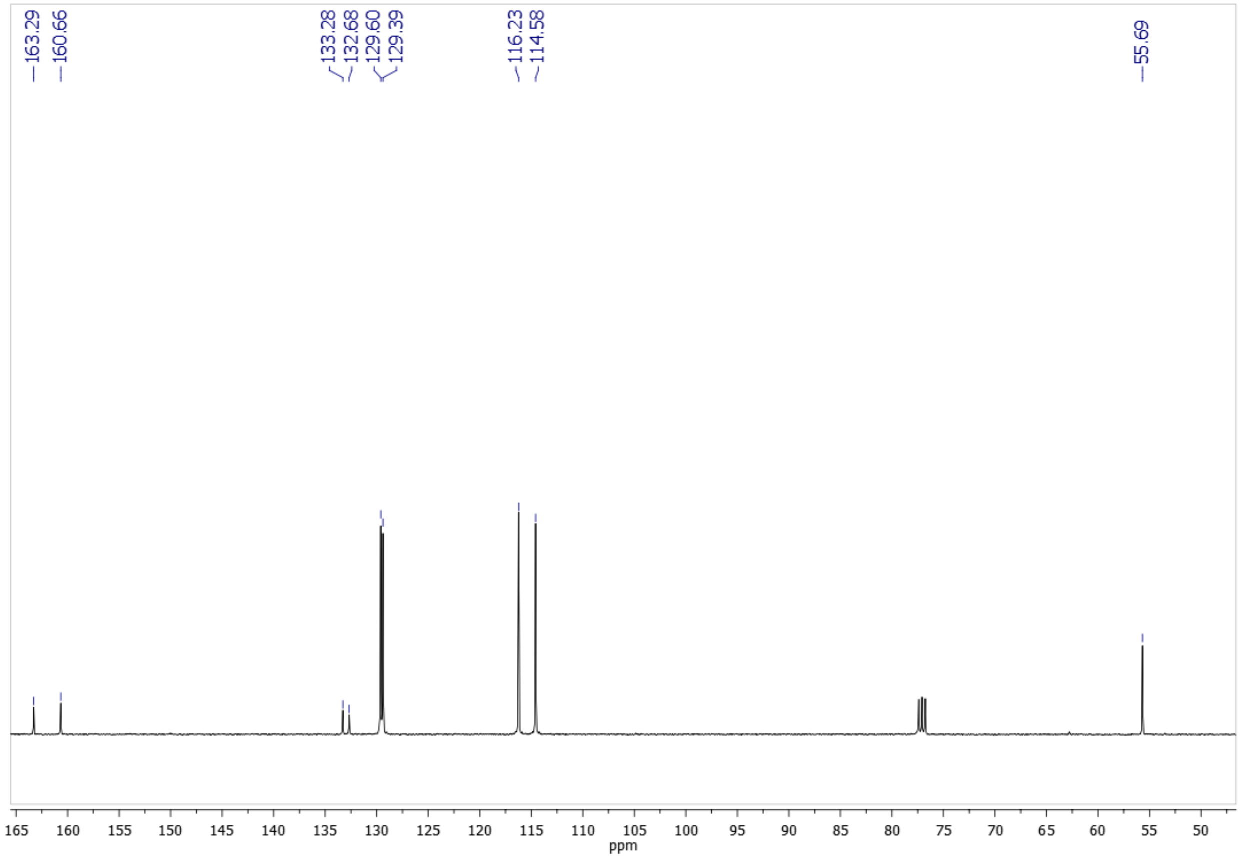

Figure S7. IR spectrum of 4-((4-methoxyphenyl)sulfonyl)phenol

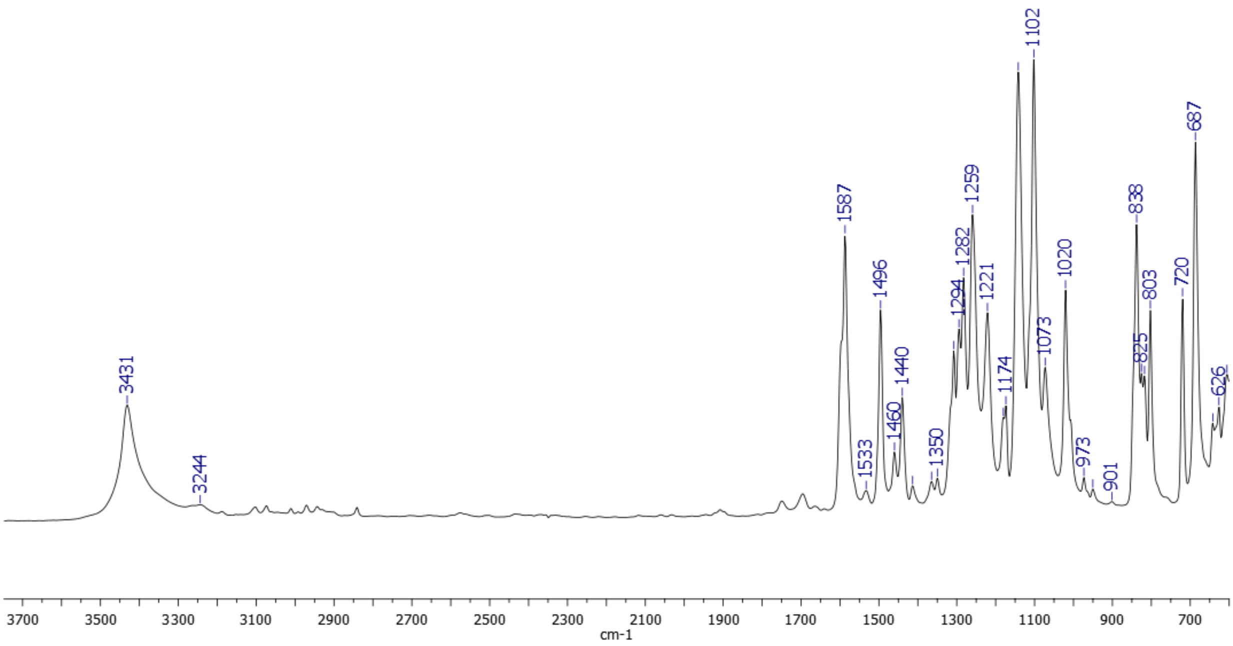

Figure S8. EI mass spectrum of 4-((4-methoxyphenyl)sulfonyl)phenol

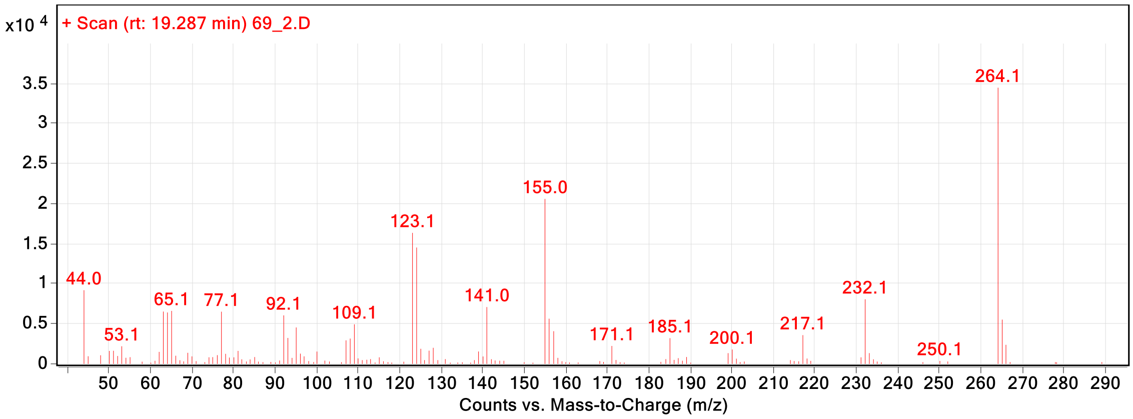

Figure S9. NMR ^{1}H spectrum of (4-cyanatophenyl)(4-methoxyphenyl)sulfane

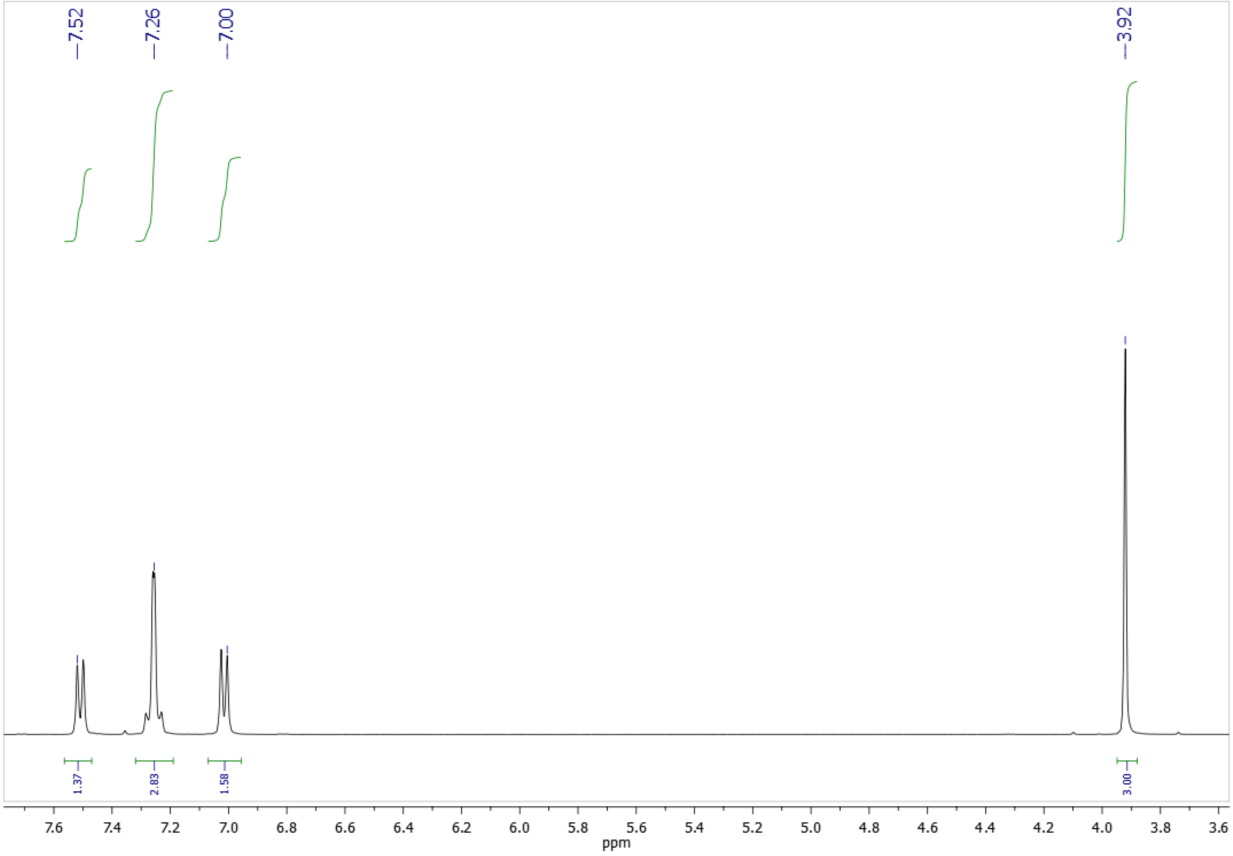

Figure S10. NMR ^{13}C spectrum of (4-cyanatophenyl)(4-methoxyphenyl)sulfane

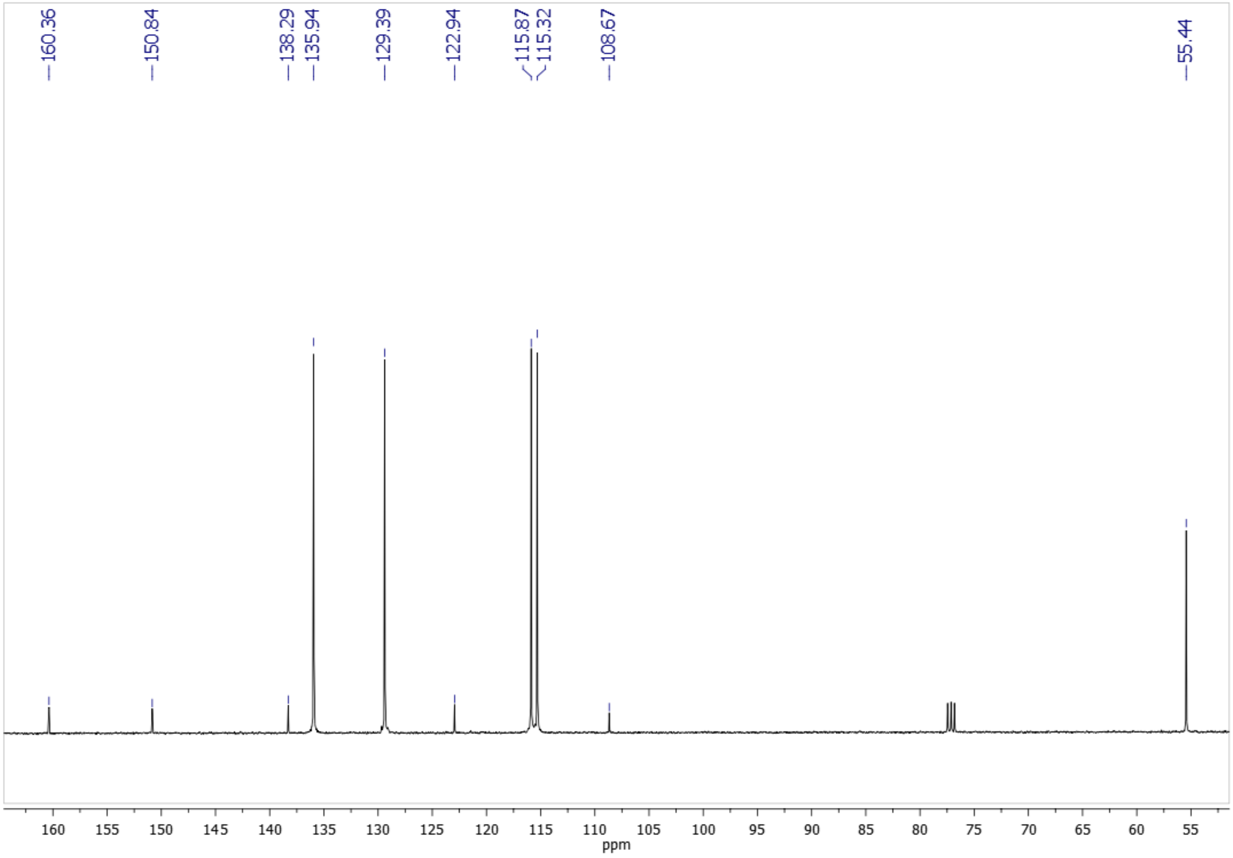

Figure S11. IR spectrum of (4-cyanatophenyl)(4-methoxyphenyl)sulfane

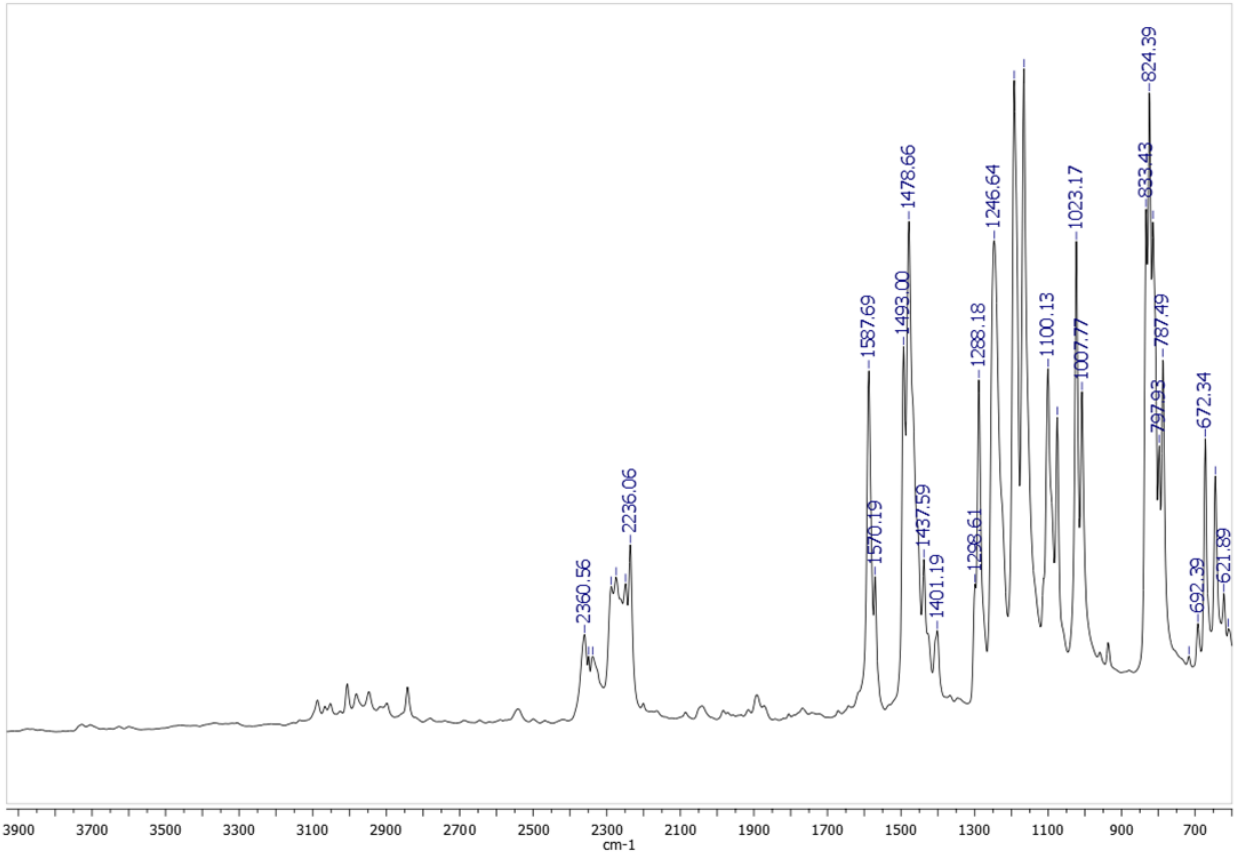

Figure S12. EI mass spectrum of (4-cyanatophenyl)(4-methoxyphenyl)sulfane

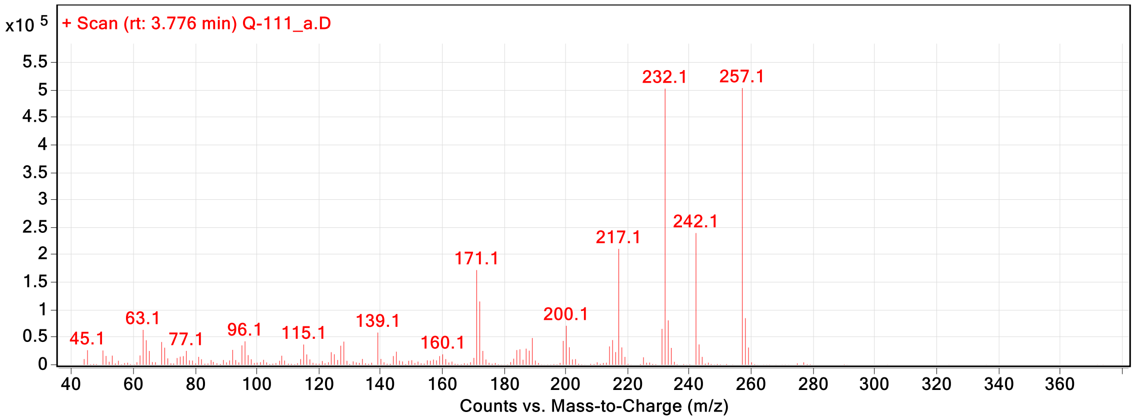

Figure S13. MALDI mass spectrum of (4-cyanatophenyl)(4-methoxyphenyl)sulfane cyclotrimerization product

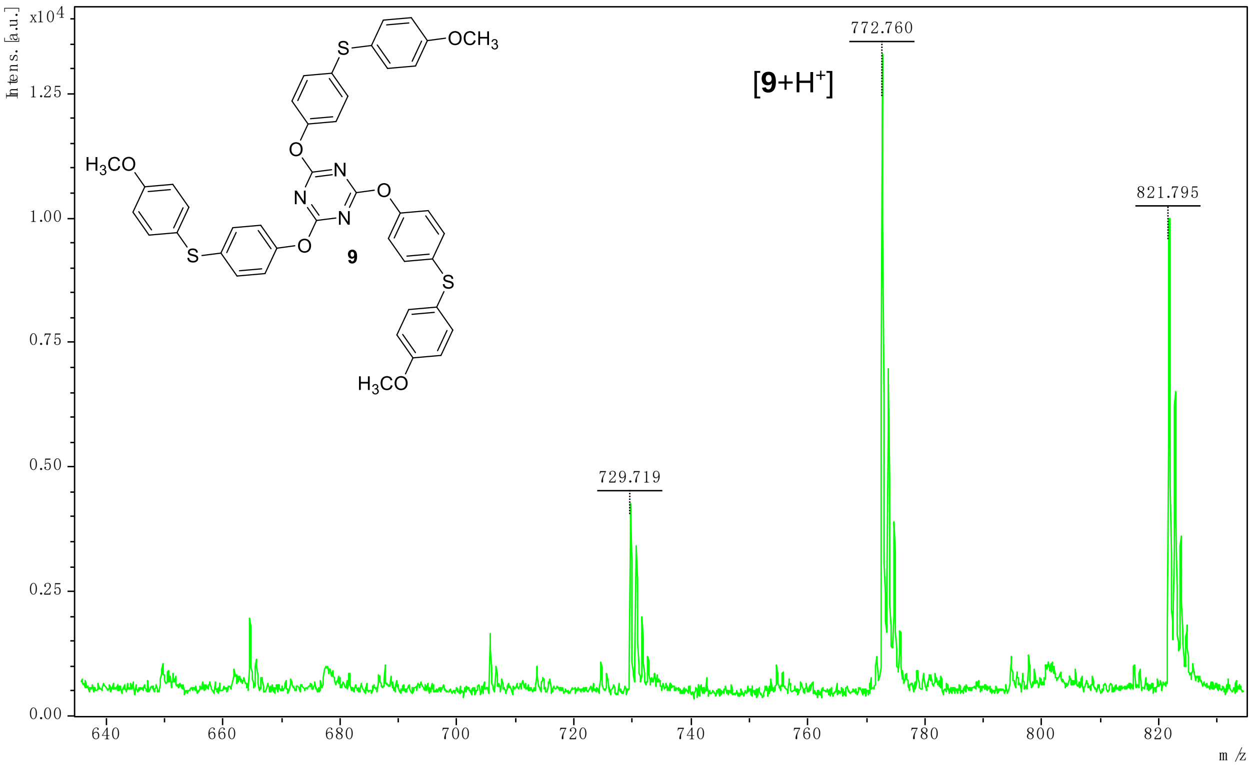

Supplement: Supplementary file 1 [file molecules-24-00177-s001.pdf]
